# Methodological and reporting quality of machine learning studies on cancer diagnosis, treatment, and prognosis

**DOI:** 10.3389/fonc.2025.1555247

**Published:** 2025-04-14

**Authors:** Aref Smiley, David Villarreal-Zegarra, C. Mahony Reategui-Rivera, Stefan Escobar-Agreda, Joseph Finkelstein

**Affiliations:** ^1^ Department of Biomedical Informatics, University of Utah, Salt Lake City, UT, United States; ^2^ Telehealth Unit, Universidad Nacional Mayor de San Marcos, Lima, Peru

**Keywords:** cancer, artificial intelligence, diagnosis, prognosis, therapy

## Abstract

This study aimed to evaluate the quality and transparency of reporting in studies using machine learning (ML) in oncology, focusing on adherence to the Consolidated Reporting Guidelines for Prognostic and Diagnostic Machine Learning Models (CREMLS), TRIPOD-AI (Transparent Reporting of a Multivariable Prediction Model for Individual Prognosis or Diagnosis), and PROBAST (Prediction Model Risk of Bias Assessment Tool). The literature search included primary studies published between February 1, 2024, and January 31, 2025, that developed or tested ML models for cancer diagnosis, treatment, or prognosis. To reflect the current state of the rapidly evolving landscape of ML applications in oncology, fifteen most recent articles in each category were selected for evaluation. Two independent reviewers screened studies and extracted data on study characteristics, reporting quality (CREMLS and TRIPOD+AI), risk of bias (PROBAST), and ML performance metrics. The most frequently studied cancer types were breast cancer (n=7/45; 15.6%), lung cancer (n=7/45; 15.6%), and liver cancer (n=5/45; 11.1%). The findings indicate several deficiencies in reporting quality, as assessed by CREMLS and TRIPOD+AI. These deficiencies primarily relate to sample size calculation, reporting on data quality, strategies for handling outliers, documentation of ML model predictors, access to training or validation data, and reporting on model performance heterogeneity. The methodological quality assessment using PROBAST revealed that 89% of the included studies exhibited a low overall risk of bias, and all studies have shown a low risk of bias in terms of applicability. Regarding the specific AI models identified as the best-performing, Random Forest (RF) and XGBoost were the most frequently reported, each used in 17.8% of the studies (n = 8). Additionally, our study outlines the specific areas where reporting is deficient, providing researchers with guidance to improve reporting quality in these sections and, consequently, reduce the risk of bias in their studies.

## 1 Introduction

Cancer is one of the leading causes of disease burden and mortality worldwide. Early detection is crucial for improving clinical outcomes, yet approximately 50% of cancers are diagnosed at an advanced stage (1). Artificial Intelligence (AI), particularly machine learning (ML) models, has shown great potential in cancer diagnosis, prognostic, and treatment, standing out for its high accuracy, sensitivity, and ability to integrate into clinical workflows (2). This progress has led to a significant increase in studies and publications dedicated to the development and evaluation of these models in recent years (3).

The quality of reporting in these studies is critical, as clear and comprehensive documentation allows for the validation of results and their replication in different contexts (2). However, growing concerns have emerged regarding the completeness and accuracy of reports in ML-based research. Indeed, a meta-review of fifty systematic reviews, revealed that most reports of primary diagnostic accuracy studies were incomplete, limiting their utility and reproducibility (4). Furthermore, systematic reviews on treatment response in cancer patients have identified high heterogeneity and reporting issues (5, 6).

In response to these deficiencies, specific guidelines, such as the Consolidated Reporting Guidelines for Prognostic and Diagnostic Machine Learning Models (CREMLS) and the Transparent Reporting of a Multivariable Prediction Model for Individual Prognosis or Diagnosis (TRIPOD+AI), have been developed to standardize the key aspects that should be included in ML study reports (7, 8). Nevertheless, the widespread adoption of these tools depends on their dissemination and proper training, which may delay their integration into recent scientific publications (9). In addition to ensuring the quality of reporting, assessing the risk of bias in studies using AI models for cancer patients is essential. The Prediction Model Risk of Bias Assessment Tool (PROBAST) is a tool designed to evaluate the risk of bias across four domains: participants, predictors, outcomes, and analysis (10).

Our study evaluated PubMed publications that applied ML for the diagnosis, treatment, and prognosis of cancer patients. We assessed reporting quality (CREMLS and TRIPOD+AI), risk of bias (PROBAST), and ML performance metrics.

## 2 Materials and methods

### 2.1 Eligibility criteria

We included primary studies that applied AI techniques to predict cancer diagnosis, treatment, and prognosis in patients. Studies were eligible regardless of participants’ age, sex, race, ethnicity, or other sociodemographic or clinical characteristics. Articles written in any language and employing observational or experimental study designs were considered. Reviews, viewpoints, and conference papers were excluded. Our study included research that evaluates the first case of a specific cancer, specific recurrent cancers, groups of cancers, or metastases.

### 2.2 Search strategy and sources

Our search strategy included terms related to oncology, AI, prognosis, diagnosis, and treatment. A search was conducted in PubMed, restricted to studies published between February 1, 2024, and January 31, 2025. The results were sorted by “most recent,” and the 15 most recent studies on prognosis, diagnosis, and treatment were selected, yielding a total of 45 articles. The most recent articles were chosen in order to reflect the current state of the rapidly evolving landscape of AI applications in oncology. Details of the search strategy are provided in **Supplementary Material 1**.

### 2.3 Selection process

The selection process was conducted using Rayyan (11). Two reviewers independently screened the title and abstract of each record identified to determine if the inclusion criteria were met. Subsequently, both reviewers independently evaluated the full text of records that passed the title and abstract screening. In cases of disagreement, a third reviewer made the final decision regarding inclusion.

### 2.4 Data collection process and data items

Data extraction was performed using a standardized Excel form. Two independent reviewers conducted the data collection process, and a third reviewer made the final decision in cases of disagreement. Each reviewer independently collected information using this form, which captured details such as the name of the first author, year of publication, article title, patient characteristics, type of AI tested or developed, clinical contexts, reported outcomes (AI performance metrics), reporting quality (CREMLS and TRIPOD+AI) (7, 8), and risk of bias (PROBAST) (10).

The extraction process was conducted in duplicate, and the level of agreement between independent evaluators was assessed in the first five included studies. The extracted information was highly consistent, achieving an agreement level above 80%.

### 2.5 Synthesis methods

#### 2.5.1 Report quality

The TRIPOD+AI checklist consists of 27 key items designed to ensure the quality and transparency of reporting in prediction model studies (8). This updated version of TRIPOD extends the recommendations to include models developed using ML methods. The primary objective of TRIPOD+AI is to promote clear, comprehensive, and reproducible reporting in studies that develop or validate prediction models in healthcare. TRIPOD+AI results were categorized using the following response options: Yes, No/Not reported, or Not applicable.

The CREMLS guideline was developed to enhance the transparency and quality of reporting in ML studies for diagnostic and prognostic applications in healthcare (7). It comprises 37 items organized into five categories: study details, data, methodology, model evaluation, and model explainability. CREMLS results were assessed using the following response options: Yes, No/Not reported, or Not applicable.

Since our objective was to assess the reporting quality of studies using ML for diagnostic, prognostic, and treatment outcomes in cancer patients, no studies were excluded based on a negative evaluation of reporting quality.

#### 2.5.2 Risk of bias

The PROBAST tool assessed the risk of bias (RoB) in studies on prediction models for diagnosis, prognosis, and treatment. PROBAST provides a structured framework for evaluating the internal and external validity of studies by identifying potential biases in participant selection, predictor and outcome measurement, analytical strategies, and data presentation (10).

The response options in PROBAST were: low RoB/low concern regarding applicability (+), high RoB/high concern regarding applicability (-), and unclear RoB/unclear concern regarding applicability (?).

#### 2.5.3 AI performance metrics

The study presented the main metrics used to evaluate the performance of each AI model included in the analyzed articles across different phases (i.e., training, testing, validation). These metrics included sensitivity (also known as recall or true positive rate), specificity (true negative rate), overall accuracy (probability of correct classification), positive predictive value (precision), F1 score, and the area under the ROC curve (AUC).

### 2.6 Statistical analysis

All statistical analyses were performed using R. Our study employed a descriptive approach based on percentages and absolute frequencies of CREMLS, TRIPOD+AI, and PROBAST scores. No inferential approaches focused on hypothesis testing were used.

## 3 Results

### 3.1 Study selection

**Figure 1:**
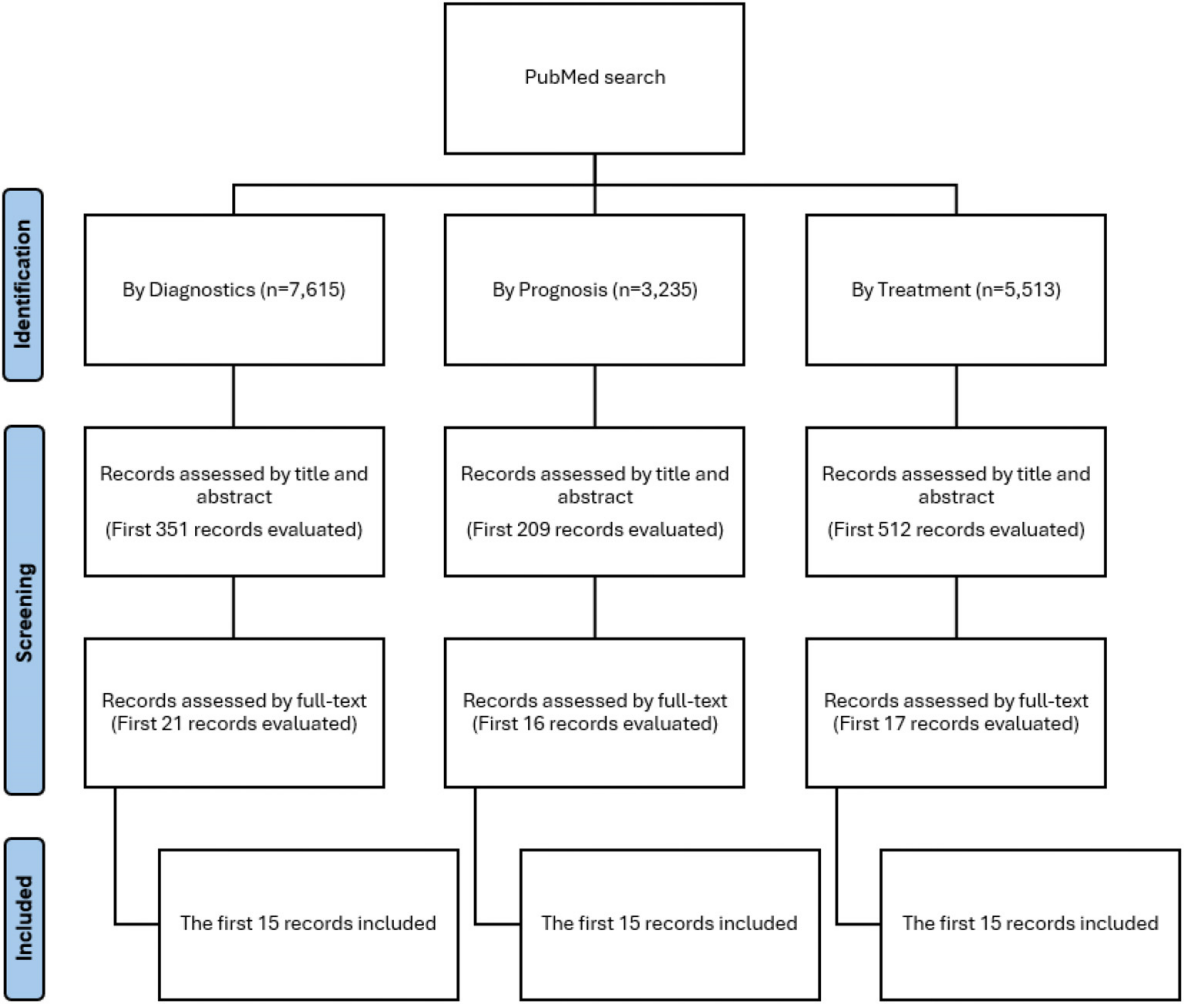
Flowchart.

Our study searched PubMed for ML models related to diagnosis, treatment, and prognosis that met the inclusion criteria. To standardize selection, we included only the first 15 articles for each category: prognosis, diagnosis, and treatment. The screening process, which involved title and abstract review followed by full-text assessment, is shown in **Figure 1**.

### 3.2 Characteristics of the included studies

The first authors of the included studies were affiliated with institutions from 16 different countries. China had the highest number of publications (n=27/45; 60%), followed by Egypt (n=3/45; 6.7%). The most frequently studied cancer types were breast cancer (n=7/45; 15.6%), lung cancer (n=7/45; 15.6%), and liver cancer (n=5/45; 11.1%). More than half of the studies did not specify the source of funding (n=25/45; 55.6%). The individual characteristics of each study are summarized in **Supplementary Table 1**. The list of included articles is presented in **Supplementary Table 2**.

### 3.3 Report quality by CREMLS

Our study found that, in the study design section, all evaluated studies adequately reported the medical/clinical task of interest, research question, overall study design, and intended use of the ML model. However, only 51% of the studies provided information on whether existing model performance benchmarks for this task were considered.

In the data section, all studies reported information on methods of data collection and data characteristics. However, a large proportion did not report details on sample size calculation (98%), known quality issues with the data (69%), or bias introduced due to the data collection method used (62%).

In the methodology section, all studies reported the rationale for selecting the ML algorithm, and 98% described the method used to evaluate model performance during training. However, no studies reported strategies for handling outliers, and the majority did not provide information on strategies for model pre-training (92%) or data augmentation (79%).

In the evaluation section, nearly all studies reported some type of performance metrics used to evaluate the model (98%) and the results of internal validation (98%). However, no studies provided information on characteristics relevant for detecting data shift and drift, and 93% did not report or discuss the cost or consequences of errors.

**Figure 2:**
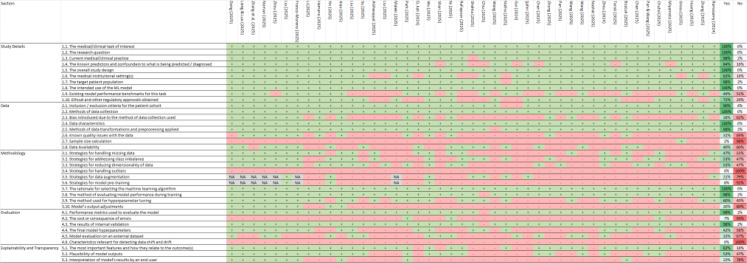
CREMLS checklist. +, Yes; -, No/No reported/The report is not clear; NA, Not applicable.

In the explainability and transparency section, 82% of the studies reported the most important features and their relationship to the outcomes. Compliance with different criteria for each individual study is detailed in **Figure 2**.

### 3.4 Report quality by TRIPOD+AI

**Figure 3:**
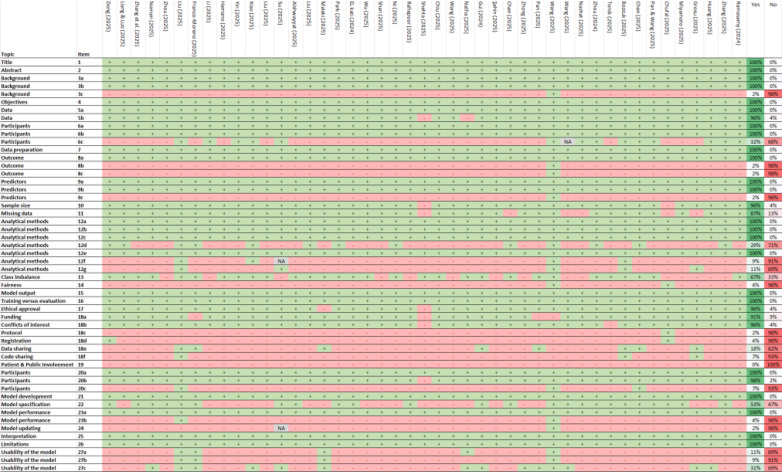
TRIPOD+AI checklist. +, Yes; -, No/No reported/The report is not clear; NA, Not applicable. 1) Identify the study as developing or evaluating the performance of a multivariable prediction model, the target population, and the outcome to be predicted. 2) See TRIPOD+AI for Abstracts checklist. 3a) Explain the healthcare context, rationale for developing or evaluating the prediction model, including references to existing models. 3b) Describe the target population and intended purpose of the prediction model, including intended users. 3c) Describe any known health inequalities between sociodemographic groups. 4) Specify the study objectives, including whether it describes development or validation of a prediction model. 5a) Describe sources of data separately for development and evaluation datasets, rationale for using these data. 5b) Specify dates of collected participant data, including start and end of participant accrual. 6a) Specify key elements of the study setting including number and location of centers. 6b) Describe the eligibility criteria for study participants. 6c) Give details of any treatments received and how they were handled during model development or evaluation. 7) Describe any data pre-processing and quality checking across sociodemographic groups. 8a) Clearly define the outcome being predicted, time horizon, and assessment method. 8b) Describe qualifications and demographic characteristics of the outcome assessors. 8c) Report any actions to blind assessment of the outcome. 9a) Describe the choice of initial predictors and any pre-selection before model building. 9b) Clearly define all predictors, including how and when measured. 9c) Describe qualifications and demographic characteristics of predictor assessors. 10) Explain how study size was determined, including sample size calculation. 11) Describe how missing data were handled and reasons for omitting data. 12a) Describe how data were used for development and evaluation of model performance. 12b) Describe how predictors were handled in the analyses. 12c) Specify model type, rationale, steps for building, hyperparameter tuning, and validation. 12d) Describe how heterogeneity in estimates and model performance was handled. 12e) Specify all measures and plots used to evaluate model performance. 12f) Describe any model updating arising from evaluation. 12g) Describe how model predictions were calculated. 13) Describe methods for handling class imbalance and recalibrating predictions. 14) Describe approaches used to address model fairness and their rationale. 15) Specify the output of the prediction model and rationale for classification thresholds. 16) Identify differences between development and evaluation data. 17) Name the ethics committee that approved the study and describe consent procedures. 18a) Give the source of funding and role of the funders. 18b) Declare any conflicts of interest and financial disclosures. 18c) Indicate where the study protocol can be accessed or state if unavailable. 18d) Provide registration information or state if not registered. 18e) Provide details of data availability. 18f) Provide details of analytical code availability. 19) Provide details of patient and public involvement or state no involvement. 20a) Describe the flow of participants through the study, including follow-up time. 20b) Report characteristics overall and by data source, including key demographics. 20c) Compare development data with evaluation data for key predictors. 21) Specify the number of participants and outcome events in each analysis. 22) Provide full details of the prediction model, including accessibility. 23a) Report model performance estimates with confidence intervals. 23b) Report results of heterogeneity in model performance. 24) Report results from model updating, including updated model. 25) Provide overall interpretation of results, including fairness. 26) Discuss study limitations, biases, and generalizability. 27a) Describe handling of poor-quality or unavailable input data. 27b) Specify required user interaction and expertise. 27c) Discuss future research steps and generalizability.

The included studies demonstrate high compliance with key reporting standards for predictive modeling. All studies (100%) clearly identified their nature as predictive modeling investigations, specified their objectives, and provided detailed descriptions of data sources and participant eligibility (**Figure 3**). Also, they precisely defined predictors and outcomes, justifying their selection and evaluation methodology. Data preparation and model validation were also well-documented, with comprehensive descriptions of analytical methods, including model type, hyperparameter tuning, and performance metrics. Furthermore, all studies addressed the interpretation of results and discussed limitations, biases, and generalizability. Ethical considerations were reported in most cases, with 96% of studies indicating approval from an ethics committee or an equivalent review process. The flow of participants and differences between development and evaluation datasets were also consistently documented.

Despite strong adherence to several TRIPOD+AI criteria, significant reporting gaps were identified. 98% of studies did not report potential health inequalities among sociodemographic groups or describe the demographic characteristics and qualifications of outcome and predictor assessors. Additionally, 98% of studies failed to indicate where the study protocol could be accessed, and 96% did not provide information on study registration or describe methods to address model equity or justify their approach. The availability of analytical code was also limited, with 93% of studies not providing details on access. A critical issue was the complete absence of patient and public involvement in study development (100%). Furthermore, 93% of studies did not compare development and evaluation data for key predictors, while 96% did not report heterogeneity in model performance. Lastly, 98% of studies did not present results on model updating, and 91% did not specify the level of user interaction or expertise required for model implementation.

### 3.5 Risk of bias by PROBAST

The methodological quality assessment using PROBAST revealed that 89% of the included studies exhibited a low overall risk of bias, suggesting adequate internal validity in most evaluated studies (see **Table 1**). Additionally, all studies demonstrated a low risk of bias in terms of applicability, indicating that the assessed predictive models are potentially transferable to real-world clinical settings.

**Table 1:** Risk of Bias by PROBAST.

First author (year)	ROB				Applicability			Overall	
	Participants	Predictors	Outcome	Analysis	Participants	Predictors	Outcome	ROB	Applicability
Dong (2025)	Low	Unclear	Low	Low	Low	Unclear	Low	Low	Low
Liang & Luo (2025)	Low	Low	Low	Low	Low	Low	Low	Low	Low
Zhang et al. (2025)	Low	Low	Low	Low	Low	Low	Low	Low	Low
Noman (2025)	Low	Low	Low	Low	Low	Low	Low	Low	Low
Zhou (2025)	Low	Unclear	Low	Low	Low	Unclear	Low	Low	Low
Liu (2025)	Low	Low	Low	Unclear	Low	Low	Low	Low	Low
Franco-Moreno (2025)	Low	Unclear	Low	Unclear	Low	Unclear	Low	Unclear	Low
Li (2025)	Low	Low	Low	Low	Low	Low	Low	Low	Low
Hamano (2025)	Low	Low	Low	Low	Low	Low	Low	Low	Low
Yin (2025)	Low	Low	Low	Low	Low	Low	Low	Low	Low
Xiao (2025)	Low	Low	Low	Low	Low	Low	Low	Low	Low
Liu (2025)	Low	Unclear	Low	Low	Low	Unclear	Low	Low	Low
Su (2025)	Low	Unclear	Low	Low	Low	Unclear	Low	Low	Low
Alshwayyat (2025)	Low	Low	Low	Low	Low	Low	Low	Low	Low
Liu (2025)	Low	Low	Low	Low	Low	Low	Low	Low	Low
Maleki (2025)	Low	Unclear	Low	Low	Low	Unclear	Low	Low	Low
Park (2025)	Low	Low	Low	Low	Low	Low	Low	Low	Low
EL kati (2024)	Unclear	Low	Low	Unclear	Low	Low	Low	Unclear	Low
Wu (2025)	Low	Low	Low	High	Low	Low	Low	High	Low
Shan (2025)	Low	Low	Low	High	Low	Low	Low	High	Low
Ni (2025)	Low	Low	Low	Low	Low	Low	Low	Low	Low
Rafiepoor (2025)	Low	Low	Low	Low	Low	Low	Low	Low	Low
Shehta (2025)	Low	Low	Low	Low	Low	Low	Low	Low	Low
Chiu (2025)	Low	Low	Low	Unclear	Low	Low	Low	Low	Low
Wang (2025)	Low	Unclear	Low	High	Low	Unclear	Low	High	Low
Natha (2025)	Low	Low	Low	Low	Low	Low	Low	Low	Low
Gui (2024)	Low	Unclear	Low	Low	Low	Unclear	Low	Low	Low
Şahin (2025)	Low	Unclear	Low	Low	Low	Unclear	Low	Low	Low
Chen (2025)	Low	Unclear	Low	Low	Low	Unclear	Low	Low	Low
Zhong (2025)	Low	Unclear	Low	Low	Low	Unclear	Low	Low	Low
Pan (2025)	Low	Low	Low	Unclear	Low	Low	Low	Low	Low
Wang (2025)	Low	Low	Low	Low	Low	Low	Low	Low	Low
Wang (2025)	Low	Low	Low	Low	Low	Low	Low	Low	Low
Nashat (2025)	Low	Low	Low	Unclear	Low	Low	Low	Low	Low
Zhou (2024)	Low	Low	Low	Unclear	Low	Low	Low	Low	Low
Torok (2025)	Low	Low	Low	Low	Low	Low	Low	Low	Low
Bozcuk (2025)	Low	Unclear	Low	Low	Low	Unclear	Low	Low	Low
Chen (2025)	Low	Low	Low	Low	Low	Low	Low	Low	Low
Pan & Wang (2025)	Low	Low	Low	Low	Low	Low	Low	Low	Low
Chufal (2025)	Low	Low	Low	Unclear	Low	Low	Low	Low	Low
Miyamoto (2025)	Low	Unclear	Low	Low	Low	Unclear	Low	Low	Low
Grosu (2025)	Low	Low	Unclear	Low	Low	Low	Unclear	Low	Low
Huang (2025)	Low	Low	Low	Low	Low	Low	Low	Low	Low
Zhang (2025)	Low	Low	Low	Low	Low	Low	Low	Low	Low
Ramasamy (2024)	Low	Low	Low	Low	Low	Low	Low	Low	Low

ROB, risk of bias; Low, indicates low ROB/low concern regarding applicability; High, indicates high ROB/high concern regarding applicability; Unclear, indicates unclear ROB/unclear concern regarding applicability.

At the domain level, the risk of bias in the participant and outcome domains was minimal, with 98% of the studies showing a low risk of bias in these areas. Regarding applicability, 100% of the studies reported low concern for the applicability of participants, while 98% indicated low concern for the applicability of outcomes.

However, the predictor domain showed an unclear risk of bias in 29% of the studies, both in terms of bias risk assessment and applicability. Furthermore, in the analysis domain, only 76% of the studies exhibited a low risk of bias, suggesting that a significant proportion may have employed methodological practices that compromise the validity of their findings. Issues such as handling of missing data, predictor selection, and correction for overfitting require further attention to enhance the robustness of predictive models in future studies.

### 3.6 AI performance metrics

In terms of the AI model family used, 73.3% of the studies employed Supervised ML models (n = 33), while 24.4% used Deep Learning models (n = 11). Regarding the specific AI models identified as the best-performing, Random Forest (RF) and XGBoost were the most frequently reported, each used in 17.8% of the studies (n = 8). The performance metrics for each study are presented in **Table 2**.

**Table 2:** AI performance metrics by study.

First author (year)	Type	Type of data	Type of AI tested or developed	AI models used	Best model	AI metrics
Dong (2025)	Prognosis	Cervical screening data, including hrHPV full genotyping, cytology results, and gynecological examination findings.	Supervised Machine Learning	XGBoost, Support Vector Machine (SVM), Random Forest (RF), Naïve Bayes (NB)	XGBoost	AUROC: 0.989 (maximum), 0.781 (minimum); Brier Score: 0.118 (maximum), 0.01 (minimum)
Liang & Luo (2025)	Prognosis	Structured data	Supervised Machine Learning	XGBoost, MLP, KNN, Random Forest (RF), Logistic Regression, AJCC Staging	XGBoost	AUC (Training: 0.95, Validation: 0.78)
Zhang et al. (2025)	Prognosis	Structured data (clinical, lab results)	Supervised Machine Learning	Logistic Regression, KNN, RF, SVM, XGBoost, LightGBM	Logistic Regression	Accuracy: 0.765 (LR), Precision: 0.750 (LR), Recall: 0.710 (LR), F1-Score: 0.695 (LR), AUC: 0.812 (LR)
Noman (2025)	Prognosis	Clinical and pathological features (tumor grade, ER status, HER2, lymph node involvement, tumor size, survival status).	Supervised Machine Learning	LightGBM, XGBoost, Random Forest, Support Vector Machine (SVM), Neural Networks (NN), K-Nearest Neighbors (KNN)	LightGBM (for recurrence), XGBoost (for recurrence type differentiation)	C-index: 0.837 (survival analysis), AUC: 92% (LightGBM for recurrence), Accuracy: 86% (XGBoost for recurrence type classification)
Zhou (2025)	Prognosis	Clinical, demographic, psychological, and health-related data.	Supervised Machine Learning	Logistic Regression (LR), Support Vector Machine (SVM), Random Forest (RF), Extreme Gradient Boosting (XGBoost), Gradient Boosted Trees (GBDT), Multi-Layer Perceptron (MLP)	Random Forest (RF)	AUC: 0.925, Accuracy: 84.7%, Sensitivity: 90.5%, Specificity: 80.9%, Brier Score: 0.107
Liu (2025)	Prognosis	Pathomic features extracted from whole slide imaging (WSI) histopathology.	Deep Learning, Weakly Supervised Learning	DenseNet121, ResNet50, Inception_v3, XGBoost, Random Forest, LightGBM, Cox Regression	XGBoost (for metastasis prediction)	AUC: 0.981 (training), 0.804 (testing); C-index: 0.892 (training), 0.710 (testing)
Franco-Moreno (2025)	Prognosis	Clinical and laboratory data (D-dimer, hemoglobin, cholesterol, platelets, leukocyte count, etc.)	Supervised Machine Learning	CatBoost, XGBoost, LightGBM	CatBoost	AUC-ROC: 0.86 (95% CI: 0.83–0.87), Sensitivity: 62%, Specificity: 94%, PPV: 75%, NPV: 93%
Li (2025)	Prognosis	Clinical, pathological, and molecular data (tumor stage, lymph nodes, neural invasion, Ki67, biomarkers)	Supervised Machine Learning	Random Survival Forest (RSF), Extreme Gradient Boosting (XGBoost), Decision Survival Tree (DST)	Random Survival Forest (RSF)	C-index: 0.968 (training), 0.936 (validation); AUC: 0.968 (training), 0.936 (validation); Brier Score: 0.067
Hamano (2025)	Prognosis	Clinical and laboratory data (heart rate, respiratory rate, leukocyte count, albumin, C-reactive protein, etc.)	Supervised Machine Learning	Fractional Polynomial (FP) Regression, Kernel Fisher Discriminant Analysis (KFDA), Kernel Support Vector Machine (KSVM), XGBoost	Kernel Support Vector Machine (KSVM)	AUC-ROC: 0.834
Yin (2025)	Prognosis	Computed tomography (CT) images and clinical data (tumor characteristics, biomarkers).	Deep Learning	ResNet50 + Multilayer Perceptron (MLP)	ResNet50 + MLP	AUC-ROC: 0.96 (training), 0.87 (internal testing), 0.85 (external validation)
Xiao (2025)	Prognosis	Clinical and pathological data (tumor markers, blood tests, histopathological features).	Supervised Machine Learning	Cox proportional hazards model, Decision Tree (DT), Random Forest (RF), Support Vector Machine (SVM), Extreme Gradient Boosting (XGBoost)	RFE-XGBoost	AUC-ROC: 0.89 (95% CI: 0.82–0.94), Accuracy: 0.83 (95% CI: 0.76–0.90), F1 score: 0.81 (95% CI: 0.72–0.88), Brier Score: 0.13 (95% CI: 0.09–0.17)
Liu (2025)	Prognosis	Clinical and laboratory data (blood tests, tumor characteristics, surgical variables).	Supervised Machine Learning	Extreme Gradient Boosting (XGBoost), Random Forest (RF), Support Vector Machine (SVM), k-nearest neighbor (KNN)	XGBoost	AUC-ROC: 0.939 (training), 0.876 (validation), Accuracy: 0.8908
Su (2025)	Prognosis	CT-based radiomics features and genomic data (gene expression analysis from TCGA).	Supervised Machine Learning	Cox Regression, LASSO, Random Survival Forest (RSF)	Radiomics-based Cox Regression Model	AUC-ROC: 0.899 (1-year), 0.906 (3-year), 0.869 (5-year) in the test set; C-index: 0.819 (training), 0.892 (validation), 0.851 (test)
Alshwayyat (2025)	Prognosis	Clinical and pathological data (tumor characteristics, treatment modalities, ER/PR status).	Supervised Machine Learning	Random Forest (RF), Gradient Boosting Classifier (GBC), Logistic Regression (LR), K-Nearest Neighbors (KNN), Multilayer Perceptron (MLP)	Random Forest (RF)	AUC-ROC: 0.743, Accuracy: 70.78%, Sensitivity: 94.52%
Liu (2025)	Prognosis	Clinical and demographic data (tumor characteristics, metastasis, lymph node involvement, treatment history).	Supervised Machine Learning	XGBoost, SHapley Additive exPlanations (SHAP) tool for model interpretation.	XGBoost	AUC-ROC: 0.813 (1-year), 0.738 (3-year), 0.733 (5-year) in training; 0.781, 0.785, 0.775 in validation.
Maleki (2025)	Diagnosis	Clinical and laboratory data (paraneoplastic autoantibody panels from serum and CSF)	Supervised Machine Learning	Naive Bayes	Naive Bayes	AUC-ROC: 0.9795, Sensitivity: 85.71%, Specificity: 100%, Accuracy: 97.14%, Brier Score: 0.04
Park (2025)	Diagnosis	Electronic medical records (EMR) including medications, laboratory data, and clinical procedures.	Supervised Machine Learning	Random Forest, Lasso Logistic Regression, AdaBoost, Decision Tree, Naïve Bayes, Multilayer Perceptron	Random Forest	AUROC: 0.88 (training), 0.88 (validation); Sensitivity: 62%; Specificity: 94%
EL kati (2024)	Diagnosis	Clinical and imaging data from WBCD and WDBC.	Deep Learning	Deep Neural Network (DNN) with three hidden layers, optimized with Gradient Multi-Verse Optimizer (GMVO).	GMVO-optimized DNN	Accuracy: 93.5% (WBCD), 96.73% (WDBC); Precision: 88.06% (WBCD), 93.38% (WDBC); Specificity: 93.06% (WBCD), 95.83% (WDBC); Sensitivity: 95.64% (WBCD), 98.25% (WDBC).
Wu (2025)	Diagnosis	MRI-based radiomics and deep learning features.	Deep Learning	Deep Transfer Learning (DTL) models: vgg19, GoogLeNet, Inception_v3, Vision Transformer (ViT).	vgg19-combined fusion model	AUC-ROC: 0.990 (internal test), 0.988 (external test); Accuracy: 0.935 (internal test), 0.875 (external test); F1-score: 0.937 (internal test), 0.885 (external test).
Shan (2025)	Diagnosis	Multiphasic contrast CT images (arterial, venous, and delayed phases).	Deep Learning	3D-ResUNet (coarse-to-fine segmentation network)	3D-ResUNet	Dice score: 0.8819; Precision: 0.905 (tumors >20 mm); Recall: 0.9728 (tumors >20 mm); F1-score: 0.9377 (tumors >20 mm)
Ni (2025)	Diagnosis	LDCT images, liquid biopsy (CACs), clinical variables (age, extra-thoracic cancer history, gender).	Supervised Machine Learning	Random Forest (RF), Support Vector Machine (SVM), Logistic Regression (LR), Light Gradient Boosting (LGB), Least Absolute Shrinkage and Selection Operator (LASSO).	Random Forest (RF)	AUC-ROC: 0.99 (validation cohort); Sensitivity: 92%; Specificity: 97%.
Rafiepoor (2025)	Diagnosis	MiRNA expression data from GEO datasets.	Supervised Machine Learning	Support Vector Machine (SVM), Boosted Trees (BT), Bootstrap Forest (BF), K-Nearest Neighbors (KNN), Neural Networks (NeB), LASSO Regression, Nominal Logistic, Naïve Bayes (NB).	Support Vector Machine (SVM)	AUC-ROC: 0.94, Sensitivity: 91%
Shehta (2025)	Diagnosis	Medical imaging (microscopic blood smear images)	Deep Learning	ResNetRS50, RegNetX016, AlexNet, Convnext, EfficientNet, Inception_V3, Xception, VGG19	ResNetRS50	Accuracy: 97%, Recall: 99%, F1-score: 98%
Chiu (2025)	Diagnosis	Dermoscopic images	Deep Learning	Swin Transformer, Vision Transformer (ViT), EfficientNetB5, EfficientNetV2B2, ResNet50, VGG16	Ensemble model (Swin Transformer + Vision Transformer + EfficientNetB5)	CSMUH dataset: Accuracy 97.31% (test), 99.77% (training). ISIC dataset: Accuracy 85.38% (test), 95.86% (training). False negatives reduced from 124 to 45 (ISIC dataset) and eliminated (CSMUH dataset).
Wang (2025)	Diagnosis	NBI endoscopic images and clinical data.	Supervised Machine Learning	Random Forest (RF), Support Vector Machine (SVM), Decision Tree (DT)	Random Forest (RF)	Accuracy: 96%, Precision: 90%, Recall: 100%, AUC: 0.97
Natha (2025)	Diagnosis	Dermoscopic images	Supervised Machine Learning	Random Forest (RF), Multi-layer Perceptron Neural Network (MLPN), Support Vector Machine (SVM)	Max Voting ensemble method	Accuracy: 94.70%, Precision: 93.23%, Recall: 92.54%, F1-score: 94.20%
Gui (2024)	Diagnosis	DCE-MRI images	Deep Learning	Faster R-CNN, Mask R-CNN, YOLOv9	Improved Faster R-CNN (with ROI aligning, FPN, and additional convolutional layers)	mAP: 0.752, Sensitivity: 0.950, False positive rate: 0.133
Şahin (2025)	Diagnosis	CT images	Deep Learning	You Only Look Once (YOLOv8), YOLOv5, YOLOv7	YOLOv8	Accuracy: 95.35%, Precision: 0.972, Recall: 0.919, Sensitivity: 94.74%, Specificity: 95.83%
Chen (2025)	Diagnosis	RNA-seq gene expression data from granulosa cells.	Supervised Machine Learning	LASSO Regression, Support Vector Machine Recursive Feature Elimination (SVM-RFE), XGBoost	XGBoost	AUC-ROC: 0.875 (validation cohort); AUC-ROC: 0.795 (SVM)
Zhong (2025)	Diagnosis	Label-free multiphoton imaging (MPM) data, histopathology features, clinical information.	Supervised Machine Learning	Decision Tree (DT), Multi-Layer Perceptron (MLP), Random Forest (RF), Support Vector Machine (SVM)	Multi-Layer Perceptron (MLP) - Two-stage MINT model	AUC-ROC: 0.92 (stage 1), 1.00 (stage 2); Accuracy: 89.36% (external validation)
Pan (2025)	Treatment	MRI-based radiomics and clinical data (tumor morphology, enhancement characteristics, and diffusion-weighted imaging).	Deep Learning	Logistic Regression, Support Vector Machine (SVM), Bayes, K-Nearest Neighbors (KNN), Decision Tree, Random Forest	Bayes Model integrating MRI radiomics and clinical data	AUC-ROC: 0.829, Sensitivity: 76.9%, Specificity: 83.3%
Wang (2025)	Treatment	Clinical, serological, and ultrasound imaging data.	Supervised Machine Learning	Support Vector Machine (SVM), Random Forest (RF), Decision Tree (DT), XGBoost, LightGBM, Logistic Regression, K-Nearest Neighbors (KNN), Multilayer Perceptron (MLP), Gradient Boosting Tree (GBT), Backpropagation Neural Network (BPNN), LASSO, Stepwise Regression.	SVM + RF	AUC-ROC: 0.972 (training), 0.922 (internal validation), 0.78 (external validation); Sensitivity: 85.52%, Specificity: 97.73%.
Wang (2025)	Treatment	Transcriptomic data (RNA expression) and qPCR analysis.	Supervised Machine Learning	Random-effects meta-analysis, Forward-search optimization	Meta-analysis optimized gene signature (LAMC2, TSPAN1, MYO1E, MYOF, SULF1)	AUC-ROC: 0.99 (training), 0.89 (external validation), 0.83 (peripheral blood validation)
Nashat (2025)	Treatment	Contrast-enhanced computed tomography (CECT) imaging and clinical features.	Supervised Machine Learning	Support Vector Machine (SVM), Decision Tree, K-Nearest Neighbor, Logistic Regression, Multilayer Perceptron, Random Forest	Support Vector Machine (SVM)	Accuracy: 95.24% (volumetric response), 84.13% (histologic response); Sensitivity: 95.65% (volumetric), 89.47% (histologic); Specificity: 94.12% (volumetric), 88% (histologic)
Zhou (2024)	Treatment	Blood biomarkers (C-reactive protein, neutrophil count, lactate dehydrogenase, alanine transaminase).	Supervised Machine Learning	Support Vector Machine (SVM), XGBoost, Random Forest (RF), Decision Tree (DT), Gradient-Boosted Machine (GBM), Generalized Linear Models (GLM), Least Absolute Shrinkage and Selection Operator (LASSO)	Support Vector Machine (SVM)	AUC-ROC: 0.908 (training), 0.666 (validation cohort 1), 0.776 (validation cohort 2)
Torok (2025)	Treatment	Serum N-glycome analysis using capillary electrophoresis with laser-induced fluorescence detection (CGE-LIF).	Supervised Machine Learning	Quadratic Discriminant Analysis (QDA), Random Forest, XGBoost, Neural Network, Support Vector Classifier (SVC)	Quadratic Discriminant Analysis (QDA)	AUC-ROC: 0.829 (regression), 0.8295 (progression), 0.841 (stationary)
Bozcuk (2025)	Treatment	Clinical and mutational data (age, ECOG score, mutation type, metastases, smoking status, blood biomarkers)	Reinforcement Learning	Deep Q-Network (DQN), Extra Trees Classifier (ETC)	Deep Q-Network (DQN)	AUC-ROC: 0.80 (DQN), 0.73 (ETC)
Chen (2025)	Treatment	Multi-omics data (scRNA-seq, bulk transcriptomics, GWAS)	Supervised Machine Learning	Random Survival Forest (RSF), Bayesian Deconvolution, Gene Set Variation Analysis (GSVA)	Multi-omics-driven Machine Learning Signature (MOMLS)	AUC-ROC: 0.875 (validation cohort); Kaplan-Meier survival analysis showed significant prognostic differences.
Pan & Wang (2025)	Treatment	Clinical data (immune cell profiling, HBV DNA levels, antiviral treatment status).	Supervised Machine Learning	LASSO Regression, Random Forest (RSF), XGBoost	Random Forest (RSF)	AUC-ROC: 0.961 (RSF), 0.903 (XGBoost), 0.864 (LASSO)
Chufal (2025)	Treatment	Clinical data and DIBH waveform parameters (breath-hold amplitude, duration, consistency).	Supervised Machine Learning	Gradient Boosting, LightGBM, XGBoost, CatBoost, Logistic Regression, Random Forest, K-Nearest Neighbors, Support Vector Machines, Naive Bayes	Uncalibrated Gradient Boosting Ensemble	AUC-ROC: 0.803, Recall: 0.526
Miyamoto (2025)	Treatment	CT radiomics features (texture analysis, intensity statistics, shape features).	Supervised Machine Learning	Random Forest (RF), Boruta Algorithm	Random Forest (RF)	AUC-ROC: 0.94 (training), 0.87 (validation)
Grosu (2025)	Treatment	CT colonography images, radiomics features.	Supervised Machine Learning	Random Forest (RF)	Random Forest (RF)	Accuracy: 84% (AI-assisted), Sensitivity: 85%, Specificity: 82%, Fleiss’ kappa: 0.92
Huang (2025)	Treatment	Clinical and radiotherapy data (fraction size, cumulative dose, interruptions), pathological risk factors.	Supervised Machine Learning	eXtreme Gradient Boosting (XGBoost), Cox Regression	XGBoost	Accuracy: 57.89%, Sensitivity: 57.14%, Positive Predictive Value: 44.44%, AUC-ROC: 0.58
Zhang (2025)	Treatment	Clinical and demographic data (tumor stage, histological grade, treatment history).	Deep Learning	Balanced Individual Treatment Effect for Survival data (BITES), Cox Mixtures with Heterogeneous Effects (CMHE), DeepSurv, Cox Proportional Hazards (CPH), Random Survival Forest (RSF)	Balanced Individual Treatment Effect for Survival data (BITES)	IPTW-adjusted HR: 0.84 (CRT *vs*. surgery + RT/CRT); 0.77 (surgery + CRT *vs*. surgery + RT)
Ramasamy (2024)	Treatment	CT radiomics features, clinical data (performance status, treatment site, smoking history, SBRT dose)	Supervised Machine Learning	Multi-Layer Perceptron (MLP), Support Vector Classification (SVC), Random Forest, Adaptive Boosting (AdaBoost)	Multi-Layer Perceptron (MLP) with ADASYN sampling	AUC-ROC: 0.94 (combined model), 0.95 (clinical model for early-stage patients)

## 4 Discussion

Our study included 45 studies that employed ML models for diagnostic, prognostic, and treatment outcomes in cancer patients. The findings indicate several deficiencies in reporting quality, as assessed by CREMLS and TRIPOD+AI. These deficiencies primarily relate to sample size calculation, reporting on data quality, strategies for handling outliers, documentation of ML model predictors, access to training or validation data, and reporting on model performance heterogeneity.

These reporting limitations align with the PROBAST risk of bias assessment, which identified unclear risk of bias in the predictors and analysis domains in approximately one in four included studies. Therefore, it is plausible to conclude that reporting quality is most limited in sections related to methodology, analysis, and elements that support study reproducibility (e.g., code and datasets). These limitations may introduce a higher risk of bias.

Our study aligns with previous research identifying significant issues in the quality of reporting in studies employing AI techniques to develop clinical prediction models, including the presence of “spin” practices and poor reporting standards (12). Moreover, another systematic review concluded that most studies are at high risk of bias, citing issues such as small sample sizes, inadequate handling of missing data, and lack of external validation (13). These problems are not limited to predictive models for cancer but also extend to other disciplines within clinical diagnostics, for instance, cardiology or mental health (14, 15).

The potential of AI models to predict cancer diagnoses, prognosis, and treatment are substantial, representing an opportunity to enhance healthcare access (16). While some studies have demonstrated that the diagnostic accuracy of AI models for detecting neoplasms is remarkably high, particularly in cases such as upper gastrointestinal tract cancers (17), the methodological quality of these studies remains low, with a high risk of selection bias. Additionally, other review studies evaluating the use of AI for predicting treatment and prognostic outcomes in cancer patients have also identified reporting issues and highly restricted access to data necessary for study replication (18, 19). Regarding performance metrics, the evidence highlights the need to standardize the reporting of metrics in studies due to variability in the parameters used and the inconsistent reporting of performance outcomes (20, 21).

Currently, several initiatives aim to promote the responsible use of AI models in oncology patients, such as the American Society of Clinical Oncology’s transparency principles for AI. These principles seek to enhance transparency in AI models within oncology, potentially improving their real-world application and supporting clinical decision-making (22). However, a recent review published in a journal of this scientific society found that adherence to these principles remains limited, highlighting the need to promote their implementation (23). Nevertheless, this issue does not appear to be exclusive to oncology. A systematic review identified risks of bias and reporting quality issues in ML models across various healthcare contexts, not just oncology (13). Therefore, poor reporting quality seems to be a broader issue affecting health-related disciplines in general.

Incorporating cost-related information into AI model evaluations is essential for their real-world applicability. Understanding both direct and indirect costs would facilitate the integration of these models into clinical practice by providing a clearer assessment of the financial requirements for infrastructure, staff training, and implementation logistics (24). One of the key reporting limitations identified in the included studies was the lack of information on costs and the consequences of classification errors. This may be because most studies focus primarily on accuracy and effectiveness while overlooking the associated costs, despite evidence suggesting that AI can reduce the costs of cancer diagnosis and treatment (25). Additionally, assessing the economic impact of classification errors could help balance model performance with cost-effectiveness, ensuring that AI-driven tools provide both clinical and economic value. Future studies should integrate cost analyses alongside model performance metrics to enhance the feasibility and adoption of AI in oncology.

The analysis of AI model data must be transparent, as improper handling of missing data, outliers, participant imbalance, or dimensionality reduction can introduce biases into the model. For instance, internal biases and errors during model training may lead to misclassifications, potentially resulting in biased clinical decision-making if the model is implemented in real-world settings (26). Therefore, it is essential for AI models to ensure transparency throughout this process by sharing data, code, and any other materials that enable replication (22).

The main strength of our study lies in the detailed assessment of reporting quality using the CREMLS checklist and TRIPOD+AI checklist, and the risk of bias evaluation with PROBAST. However, an important limitation of our study is that it is not a systematic review encompassing all available evidence from multiple databases, resulting in limited representativeness.

## 5 Conclusion

Our study, using CREMLS and TRIPOD+AI, identified that AI models in oncology exhibit reporting limitations, particularly in the methodology sections related to predictors and the analysis plan. These findings align with an unclear risk of bias in one out of every four included studies, as indicated by PROBAST in the Predictors and Analysis domains. Additionally, our study outlines the specific areas where reporting is deficient, providing researchers with guidance to improve reporting quality in these sections and, consequently, reduce the risk of bias in their studies.

## Supplementary material

Supplementary Material 1 Search strategy.

Supplementary Table 1 Characteristics of the included studies.

Supplementary Table 2 List of records included.
