# Supplementary material for: Methodological and reporting quality of machine learning studies on cancer diagnosis, treatment, and prognosis
Source: Front Oncol. 2025 Apr 14;15:1555247. doi: 10.3389/fonc.2025.1555247 (PMC12034563; doi:10.3389/fonc.2025.1555247)
Supplement: Supplementary Material 1 — Search strategy. [file SupplementaryFile1.docx]

**Supplementary material 1.** Search strategy.

**Source: PubMed | Date: February 10, 2025**| **Sort by: Most Recent**

**By prognostics**

| ("Neoplasms"[MeSH Terms] OR "cancer"[Title/Abstract] OR "neoplasm*"[Title/Abstract] OR "oncolog*"[Title/Abstract])    AND    ("Artificial Intelligence"[MeSH Terms] OR "Artificial Intelligence"[Title/Abstract] OR "deep learning"[Title/Abstract] OR "machine learning"[Title/Abstract] OR "supervised learning"[Title/Abstract] OR "unsupervised learning"[Title/Abstract] OR "reinforcement learning"[Title/Abstract])    AND    ("Prognosis"[MeSH Terms] OR "prognos*"[Title/Abstract] OR "predictive model"[Title/Abstract])    AND    ("2024/02/01"[Date - Publication] : "2025/01/31"[Date - Publication]) |
| --- |

**By treatment**

| ("Neoplasms"[MeSH Terms] OR "cancer"[Title/Abstract] OR "neoplasm*"[Title/Abstract] OR "oncolog*"[Title/Abstract])    AND    ("Artificial Intelligence"[MeSH Terms] OR "Artificial Intelligence"[Title/Abstract] OR "deep learning"[Title/Abstract] OR "machine learning"[Title/Abstract] OR "supervised learning"[Title/Abstract] OR "unsupervised learning"[Title/Abstract] OR "reinforcement learning"[Title/Abstract])    AND    ("Therapeutics"[MeSH Terms] OR "treatment*"[Title/Abstract] OR "therap*"[Title/Abstract] OR "intervention"[Title/Abstract])    AND    ("2024/02/01"[Date - Publication] : "2025/01/31"[Date - Publication]) |
| --- |

**By diagnosis**

| ("Neoplasms"[MeSH Terms] OR "cancer"[Title/Abstract] OR "neoplasm*"[Title/Abstract] OR "oncolog*"[Title/Abstract])    AND    ("Artificial Intelligence"[MeSH Terms] OR "Artificial Intelligence"[Title/Abstract] OR "deep learning"[Title/Abstract] OR "machine learning"[Title/Abstract] OR "supervised learning"[Title/Abstract] OR "unsupervised learning"[Title/Abstract] OR "reinforcement learning"[Title/Abstract])    AND    ("Diagnosis"[MeSH Terms] OR "diagnos*"[Title/Abstract] OR "screening"[Title/Abstract])    AND    ("2024/02/01"[Date - Publication] : "2025/01/31"[Date - Publication]) |
| --- |
