# Supplementary material for: Methodological and reporting quality of machine learning studies on cancer diagnosis, treatment, and prognosis
Source: Front Oncol. 2025 Apr 14;15:1555247. doi: 10.3389/fonc.2025.1555247 (PMC12034563; doi:10.3389/fonc.2025.1555247)
Supplement: Supplementary Table 1 — Characteristics of the included studies. [file Table1.docx]

**Supplementary Table 1.** Characteristics of the included studies.

| **First author (year)** | **Type** | **Types of data used** | Country of first author | Type of cancer | Training | Testing | Validation | Funding |
| --- | --- | --- | --- | --- | --- | --- | --- | --- |
| Dong (2025) | Prognosis | Genetic data, clinical data, demographic data | China | Cervical cancer | 254,434 women. Only female. Median: 48 years (IQR: 42–54). Cervical cancer. Data included hrHPV full genotyping, cervical cytology, and gynecological examination results. | 297,479 women. Only female. Median: 49 years (IQR: 43–55). Cervical cancer. Screening data from multiple healthcare centers in China. | Multiple cohorts: 38,500 women (External cohort 1) 1,950 women (External cohort 2) 1,590 women (External cohort 3) 779 women (External cohort 4) 4,311 women (External cohort 5). Female. Varied by cohort, with medians ranging from 37 to 53 years.. Cervical cancer. Validated in different screening settings across China. | National Natural Science Foundation of China; Major Scientific Research Program for Young and Middle-aged Health Professionals of Fujian Province, China; Fujian Province Central Government-Guided Local Science and Technology Development Project; Fujian Province’s Third Batch of Flexible Introduction of High-Level Medical Talent Teams; Fujian Provincial Natural Science Foundation of China; Fujian Provincial Science and Technology Innovation Joint Fund. |
| Liang & Luo (2025) | Prognosis | Surveillance, Epidemiology, and End Results (SEER) database (2000–2018) | China | Lung cancer | 12500 patients. 51.13% Female. Mean: 66.27 ± 9.44 years. Small cell lung cancer (SCLC). Clinical & demographic data | 3750 patients. Small cell lung cancer (SCLC). Clinical & demographic data | - | High-level Hospital Construction Project of Maoming People’s Hospital, Science and Technology Innovation Development Program of Maoming City, Medical Research Fund of Guangdong Province, Research Project of Maoming Science and Technology Bureau, Outstanding Young Talents Program of Maoming People’s Hospital |
| Zhang et al. (2025) | Prognosis | Patient records from Second Xiangya Hospital, China | China | Oral cancer | 466 patients. 89.5% Male. Mean: 54.2 ± 11.1 years. Oral cancer. Clinical & surgical data | 140 patients. Oral cancer. Clinical & surgical data | - | Not specified |
| Noman (2025) | Prognosis | Clinical and pathological data from METABRIC, MSK, Duke University, SEER, and Baheya Foundation. | Egypt | Breast cancer | 272,252 cases (combined datasets). Breast cancer. Combination of clinical, pathological, and survival data. | Breast cancer. Validation performed on real patient data from Baheya Foundation. | 468 (316 recurrence cases, 152 non-recurred cases). Breast cancer. External validation using Baheya Foundation data. | The Science, Technology & Innovation Funding Authority (STDF) in cooperation with The Egyptian Knowledge Bank (EKB). |
| Zhou (2025) | Prognosis | Clinical, demographic, psychological, and health-related data from five hospitals in China. | China | Gastric cancer | 875 patients. 71.8% male, 28.2% female. 45.1% ≥60 years, 54.9% <60 years. Gastric cancer. Factors analyzed: ethnicity, education, residence, medical insurance, medical history, family support, health literacy, psychological state. | - | 200 patients (external validation cohort). Gastric cancer. External validation performed in four independent hospitals. | National Natural Science Foundation of China (Grant No. 72304060) |
| Liu (2025) | Prognosis | Pathological images from radical mastectomy specimens at Zhejiang University Medical College. | China | Breast cancer | 204 cases (70% training set: 142 cases). 132 female (luminal), 72 female (non-luminal), 1 male (luminal). Range: 27–89 years. Breast cancer (Luminal and Non-luminal subtypes). Histopathological data including ER, PR, HER2, Ki-67 markers, tumor size, vascular invasion. | 62 cases (30% test set). Breast cancer (Luminal and Non-luminal subtypes). | - | National Natural Science Foundation of China (Grant No. 81602465) |
| Franco-Moreno (2025) | Prognosis | Clinical and laboratory data from a prospective registry in two Spanish hospitals (2005–2021). | Spain | Various (Prostate, lung, gastrointestinal, breast, hematologic, bladder) | 815 patients. 51.5% male, 48.5% female. Median: 59 years (±18). Various (Prostate: 19.6%, Lung: 10.7%, GI tract: 10.7%, Breast: 5.5%, Hematologic: 3.8%, Bladder: 3.8%). Patients with symptomatic acute VTE (DVT or PE), confirmed via imaging. | 204 patients (25% test set). Validation performed on an independent test set. | - | Not specified |
| Li (2025) | Prognosis | Clinical, pathological, and molecular data from Shanxi Cancer Hospital. | China | Gastric cancer | 405 patients (70% training set). 79.5% male, 20.5% female. Mean: 58.78 years (SD: 8.93). Gastric cancer (dMMR subtype). Clinicopathological variables included tumor stage, lymph node involvement, vascular invasion, neural invasion, Ki67, and molecular biomarkers. | 177 patients (30% test set). 80.2% male, 19.8% female. Mean: 58.47 years (SD: 9.29). Gastric cancer (dMMR subtype). | Model validation conducted using internal and external datasets. | Not specified |
| Hamano (2025) | Prognosis | Clinical and laboratory data from the J-ProVal study, covering 58 palliative care services | Japan | Various (lung, gastrointestinal, gynecological, urogenital, breast, and others) | 915 patients. 61.4% male, 38.6% female. Mean: 68.3 years (SD: 12.9). Various (Lung: 24.7%, Gastrointestinal: 27.2%, Gynecological: 5.2%, Urogenital: 4.9%, Breast: 5.2%, Other: 32.7%). Patients in palliative care with metastatic cancer; 84% had distant metastases. | - | - | Not specified |
| Yin (2025) | Prognosis | Clinical and radiomics data from Harbin Medical University Cancer Hospital and Affiliated Fourth Hospital of Harbin Medical University. | China | Liver cancer | 122 patients (92 training, 30 internal testing). 85.2% male, 14.8% female. Median: 54 years (SD: 10.2). Hepatocellular carcinoma (HCC). Clinical and imaging features including AFP levels, tumor size, vascular invasion, and radiomics data. | - | 50 patients (external validation). Hepatocellular carcinoma (HCC). External validation performed using independent dataset. | Not specified |
| Xiao (2025) | Prognosis | Clinical and pathological data from Yixing Hospital, China. | China | Colorectal cancer | 411 patients. 59.6% male, 40.4% female. Median: 64 years. Colorectal cancer (colon: 46.5%, rectal: 53.5%). Collected clinical, laboratory, and pathological data, including blood test markers and tumor staging. | - | - | Not specified |
| Liu (2025) | Prognosis | Clinical and laboratory data from multiple hospitals (Wuxi People’s Hospital and Wuxi Second People’s Hospital). | China | Colorectal cancer | 1,097 patients (internal validation: 787, external validation: 310). Colorectal cancer. Collected 34 clinical variables, including patient demographics, medical history, surgical details, and postoperative laboratory results. | - | 310 patients (external validation). Colorectal cancer. External validation performed with an independent hospital dataset. | Not specified |
| Su (2025) | Prognosis | Clinical and CT imaging data from multiple medical centers, with genomic data from TCGA. | China | Ovarian cancer | 245 patients. Ovarian cancer. Clinical data include tumor stage, histologic grade, lymphatic invasion, venous invasion, and residual disease. | 105 patients (test cohort from TCGA-OV). Ovarian cancer. Genomic analysis performed using differentially expressed genes (DEGs). | 105 patients (internal validation cohort). Ovarian cancer. Validation performed using a separate patient dataset. | Not specified |
| Alshwayyat (2025) | Prognosis | Surveillance, Epidemiology, and End Results (SEER) database. | Jordan | Breast adenoid cystic carcinoma (BACC) | 970 patients (80% training set). Median: 60 years. Breast adenoid cystic carcinoma (BACC). Clinical variables included tumor size, lymph node involvement, ER/PR status, and treatment details. | 242 patients (20% test set). Breast adenoid cystic carcinoma (BACC). | - | Not specified |
| Liu (2025) | Prognosis | Surveillance, Epidemiology, and End Results (SEER) database. | China | Renal pelvic transitional cell carcinoma (TCC) | 1,887 patients (training: 1,320; validation: 567). 57.1% male, 42.9% female. Mean: 70.7 years (SD: 11.3). Renal pelvic TCC. Collected variables included tumor size, grade, T and N staging, surgery status, lymph node involvement, metastasis sites (lung, liver, bone), and treatment modalities. | - | 567 patients (internal validation set). 57.0% male, 43.0% female. Mean: 70.9 years (SD: 11.2). Renal pelvic TCC. Model validation performed within the SEER database dataset. | Doctoral Program of the First Affiliated Hospital of Chongqing Medical University (No. CYYY-BSYJSCXXM-202332). |
| Maleki (2025) | Diagnosis | Clinical and laboratory data from University of Iowa Hospitals and Clinics, Mayo Clinic Laboratories, and ARUP Laboratories. | Iran | Various (Small cell lung cancer, prostate cancer, lung adenocarcinoma, adrenal cancer, ovarian cancer, and others) | 116 participants. 53.4% female, 46.6% male. Mean: 57.1 years, Median: 63 years, Range: 1.1–89 years. Various (Small cell lung cancer: 55%, prostate cancer: 15%, lung adenocarcinoma: 5%, adrenal cancer: 5%, ovarian cancer: 5%, others: 15%). All participants tested positive for at least one paraneoplastic autoantibody. | - | - | Self-funding |
| Park (2025) | Diagnosis | Electronic medical records (EMR) from Kyung Hee Medical Center (KHMC) and Gangdong Kyung Hee University Medical Center (KHNMC) in South Korea. | South Korea | Various (metastatic melanoma, lung cancer, renal cell carcinoma, triple-negative breast cancer) | 397 patients. 79.7% male, 20.3% female. Mean: 67.8 years (SD: 10.3). Various (metastatic melanoma, lung cancer, renal cell carcinoma, triple-negative breast cancer). Patients treated with PD-1 (nivolumab, pembrolizumab) or PD-L1 (atezolizumab) inhibitors. | 255 patients. 60.2% male, 39.8% female. Mean: 66.2 years (SD: 9.8). Various. Independent validation using an external cohort. | Various. Model validation conducted via cross-comparison between two independent hospital datasets. | Not specified |
| EL kati (2024) | Diagnosis | Clinical and imaging data from Wisconsin Breast Cancer Dataset (WBCD) and Wisconsin Diagnosis Breast Cancer (WDBC). | China | Breast cancer | 699 (WBCD), 569 (WDBC). Only female. Breast cancer. WBCD consists of 458 benign and 241 malignant cases, while WDBC consists of 357 benign and 212 malignant cases. | 30% of dataset (210 for WBCD, 171 for WDBC). Breast cancer. | - | National Natural Science Foundation of China (Grant Nos. 61672011 and 61472467). |
| Wu (2025) | Diagnosis | Contrast-enhanced MRI (CE-MRI) data from four medical centers. | China | Liver cancer | 244 patients (training set). 179 males, 65 females. Median: 60 years. 81 DPHCC, 87 HCC, 76 ICC. Clinical data included age, sex, cirrhosis status, viral hepatitis markers, AFP, AST, ALT, and tumor size. | 62 patients (internal test set). 43 males, 19 females. Median: 62 years. 21 DPHCC, 21 HCC, 20 ICC. | 75 patients (external test set). 45 males, 30 females. Median: 63 years. 30 HCC, 20 DPHCC, 25 ICC. External validation performed using an independent dataset from two additional hospitals. | Suzhou Science and Technology Bureau Project (SKY2023146, SSD2024083); China Postdoctoral Science Foundation General Program (2024M752334); Jiangsu Provincial Medical Key Discipline Cultivation Unit (JSDW202242). |
| Shan (2025) | Diagnosis | Multiphasic contrast CT images from a clinical dataset. | China | Liver cancer | 140 patients. 121 male (86.4%), 19 female (13.6%). Mean: 58 years (SD: 11). Hepatocellular carcinoma (HCC). CT imaging dataset; lesions segmented and confirmed by expert radiologists using InferScholar software. | - | Hepatocellular carcinoma (HCC). Subgroup analysis performed for lesion size and location. | National Natural Science Foundation of China (Grant No. 82271993). |
| Ni (2025) | Diagnosis | LDCT images, liquid biopsy (CACs), and clinical data from The First Affiliated Hospital of Zhengzhou University and external validation datasets. | China | Lung cancer | 971 patients. Lung cancer (non-smokers). LDCT images analyzed with AI, liquid biopsy for CAC detection, and clinical variables included. | - | 150 non-smoking patients. Lung cancer (non-smokers). External validation using an independent dataset of non-smokers. | Health Commission of Henan Province (SB201901016) |
| Rafiepoor (2025) | Diagnosis | Gene Expression Omnibus (GEO) database (7 datasets), computational bioinformatics, and machine learning models. | Iran | Skin cancer | Melanoma. MiRNA expression data analyzed from GEO datasets; selection criteria based on adjusted p-values and AUC scores. | - | Melanoma. Model validation performed using K-fold cross-validation. | Not specified |
| Shehta (2025) | Diagnosis | Dataset from Kaggle (Leukemia dataset with 15,135 images), CNMC dataset (Chinese National Medical Center). | Egypt | Blood cancer (Leukemia, Lymphoma, Multiple Myeloma) | Blood cancer (Leukemia, Lymphoma, Multiple Myeloma). Preprocessed images (224x224 pixels), augmented using rotation, flipping, and contrast stretching. | - | Blood cancer (Leukemia, Lymphoma, Multiple Myeloma). Validation performed using CNMC dataset. | Science, Technology & Innovation Funding Authority (STDF), Egyptian Knowledge Bank (EKB) |
| Chiu (2025) | Diagnosis | Dermoscopic image datasets from ISIC and Chung Shan Medical University Hospital (CSMUH). | Taiwan | Skin cancer | CSMUH dataset: 666 cases; ISIC dataset: 19,163 cases. Melanoma, Non-melanoma skin cancer, Benign lesions. CSMUH dataset: 111 melanoma cases, 199 melanocytic nevus, 45 basal cell carcinoma, 82 actinic keratosis, 134 benign keratosis, 42 dermatofibroma, 53 vascular lesions. ISIC dataset: 5008 melanoma, 6705 melanocytic nevus, 3261 basal cell carcinoma, 1043 actinic keratosis, 2657 benign keratosis, 236 dermatofibroma, 253 vascular lesions. | - | Melanoma, Non-melanoma skin cancer. Performance validation in ISIC and CSMUH datasets using a two-stage classification approach. | Not specified |
| Wang (2025) | Diagnosis | Clinical and NBI endoscopic data from Tianjin First Central Hospital, China. | China | Glottic cancer | 200 patients. 77% male, 23% female. Mean: 57.55 years (range: 27–83). Glottic cancer. Patients were classified based on laryngoscopic findings, lesion morphology, and clinical characteristics (hoarseness duration, hypertension, diabetes, smoking, alcohol consumption). | - | Glottic cancer. Model validation performed using 5-fold and 10-fold cross-validation. | Not specified |
| Natha (2025) | Diagnosis | Dermoscopic image datasets (HAM10000, ISIC 2018). | India, China, Saudi Arabia | Skin cancer | HAM10000: 10,015 cases, ISIC 2018: 13,788 cases. Melanoma, Non-melanoma skin cancer. Images labeled with 7 skin cancer types: Melanocytic nevi, Melanoma, Basal cell carcinoma, Actinic keratosis, Benign keratosis, Dermatofibroma, Vascular lesions. | - | Melanoma, Non-melanoma skin cancer. Model evaluation performed using a 10-fold cross-validation approach. | Not specified |
| Gui (2024) | Diagnosis | DCE-MRI datasets from Sun Yat-sen University Cancer Center (SYSUCC) and Duke University public dataset. | China | Breast cancer | 485 cases (internal dataset). Breast cancer. Includes malignant (219 cases) and benign (266 cases) tumors, confirmed by biopsy or surgical pathology. | - | 220 cases (public dataset from Duke University). Breast cancer. Includes malignant cases from the Duke dataset combined with benign cases from SYSUCC. | Not specified |
| Şahin (2025) | Diagnosis | CT images from Kocaeli University Faculty of Medicine, Türkiye. | Türkiye | Liver cancer | 122 patients, 1,290 CT images (training: 70%). Hepatocellular carcinoma (HCC). Patients underwent liver surgery; all cases were histopathologically confirmed. | - | 20% of dataset (internal validation). Hepatocellular carcinoma (HCC). Validation performed on a subset of the dataset. | Not specified |
| Chen (2025) | Diagnosis | RNA-seq data from granulosa cells (Second Xiangya Hospital, Central South University) and GEO datasets (GSE34526, GSE137684). | China | Polycystic ovary syndrome (PCOS) | 38 women (13 controls, 25 PCOS). Female. Polycystic ovary syndrome (PCOS). Differential gene expression analysis and immune cell infiltration assessment via CIBERSORT. | - | External validation using datasets GSE155489 (4 PCOS, 4 controls), GSE168404 (5 PCOS, 5 controls), and GSE95728 (7 PCOS, 7 controls).. Female. Polycystic ovary syndrome (PCOS). Validation performed on external datasets using XGBoost. | National Natural Science Foundation of China (No. 82201879); Hunan Provincial Natural Science Foundation (No. 2022JJ40675); Hunan Provincial Health Commission (No. B202305037231); China Postdoctoral Science Foundation. |
| Zhong (2025) | Diagnosis | Multiphoton imaging (MPM) data and histopathology data from China-Japan Friendship Hospital and Cancer Hospital, Chinese Academy of Medical Sciences. | China | Breast cancer | 50 patients (CJFH cohort: 5 benign, 15 carcinoma in situ (CIS), 30 invasive carcinoma (IC)). 100% female. ≤50 years: 40%; >50 years: 60%. Breast cancer (benign, CIS, IC). Multiphoton imaging analysis of formalin-fixed paraffin-embedded (FFPE) tissue samples. | - | 21 patients (PUCH cohort, all invasive carcinoma). 100% female. ≤50 years: 42.86%; >50 years: 57.14%. Breast cancer (all invasive carcinoma). Multiphoton imaging analysis of biopsy and surgical samples from patients receiving NAIT. | Not specified |
| Pan (2025) | Treatment | Clinical and MRI radiomics data from Affiliated Hospital of Guangdong Medical University. | China | Breast cancer | 102 patients (training: 70%, testing: 30%). Only female. Mean: 49 years (SD: 10.40), Range: 27–78 years. Breast cancer. Patients classified into LVI-positive (n=44) and LVI-negative (n=58) groups. MRI features analyzed included tumor size, axillary lymph node metastasis, peritumoral enhancement, and MRS-Cho peak. | - | Breast cancer. Model validated using internal dataset split (70% training, 30% testing). | Not specified |
| Wang (2025) | Treatment | Clinical, serological, and ultrasound imaging data from multiple hospitals (Qingdao University Affiliated Hospital, Weifang Medical University, Yantaishan Hospital). | China | Gallbladder adenomas and cholesterol polyps | 1,009 patients (training: 706, internal validation: 303). 65.24% female, 34.76% male. Mean: 52.94 years (SD: 13.42). Gallbladder adenomas and cholesterol polyps. Collected data included gallbladder wall thickness, polyp size, polyp echo, pedunculation, metabolic syndrome, adenosine deaminase (ADA), and glucose levels. | - | 139 patients (external validation set). 69% female, 31% male. Mean: 51.38 years (SD: 9.68). Gallbladder adenomas and cholesterol polyps. External validation conducted using an independent hospital dataset. | Not specified |
| Wang (2025) | Treatment | Transcriptomic datasets from GEO (14 datasets, 845 samples) and qPCR validation on peripheral blood samples. | China | Pancreatic cancer | 845 patients (from GEO datasets). Pancreatic cancer. Multi-cohort transcriptomic analysis; included tumor tissue and peripheral blood samples. | - | 55 patients (peripheral blood validation cohort). Pancreatic cancer. qPCR validation on 30 pancreatic cancer patients and 25 healthy controls. | Not specified |
| Nashat (2025) | Treatment | Clinical and CECT imaging data from Mansoura Urology and Nephrology Center, Egypt. | Egypt | Wilms tumor (WT) | 54 patients (63 tumors). 61.1% male, 38.9% female. Median: 4 years (IQR: 2.0–6.0). Wilms tumor (WT). Patients received 4–8 weeks of vincristine/actinomycin-D; tumors classified based on volumetric and histologic response. | - | Wilms tumor (WT). Model validation performed using K-fold cross-validation (LOSO, 4-fold, 10-fold). | Not specified |
| Zhou (2024) | Treatment | Blood test data from four multicenter clinical trials (OAK, BIRCH, POPLAR, FIR). | China, Germany, USA | Lung cancer | 558 patients (OAK trial). Non-small cell lung cancer (NSCLC). Patients treated with atezolizumab; 21 blood biomarkers analyzed for ML model development. | - | Validation cohort 1: 568 patients (BIRCH trial), Validation cohort 2: 193 patients (POPLAR & FIR trials combined). Non-small cell lung cancer (NSCLC). Independent validation using external trial cohorts. | National Natural Science Foundation of China (Grant No. 82060475), Chunhui Program of the Chinese Ministry of Education (Grant No. HZKY20220231), Natural Science Foundation of Guizhou Province (Grant No. ZK2022-YB632), Youth Talent Project of Guizhou Provincial Department of Education (Grant No. QJJ2022-224), China Lung Cancer Immunotherapy Research Project. |
| Torok (2025) | Treatment | Serum samples from 33 lung cancer patients undergoing chemotherapy at Borsod Academic County Hospital, Hungary. | Hungary | Lung cancer | 33 patients (98 serum samples). Mean: 63.4 years, Range: 47–74 years. Lung cancer (squamous cell carcinoma, adenocarcinoma, small cell neuroendocrine carcinoma). Serum samples collected before and after chemotherapy, analyzed using CGE-LIF. | - | Lung cancer. Validation performed using multiple iterations of model training and testing (cross-validation). | National Research, Development and Innovation Office of Hungary (Grant No. 2023-1.2.1-ERA_NET-2023-00015), Andras Koranyi Foundation, Cooperative Doctoral Program of the Ministry of Culture and Innovation, University of Debrecen Program for Scientific Publication. |
| Bozcuk (2025) | Treatment | Clinical and mutational data from 14 medical centers (retrospective study). | Turkey | Lung cancer | 318 patients. 52.2% female, 47.8% male. Median: 63 years. EGFR-mutant advanced NSCLC. 83.3% ECOG 0–1; data included TKI treatment line, EGFR mutation type, comorbidities, metastases, smoking status, and neutrophil-to-lymphocyte ratio. | - | Non-small cell lung cancer (NSCLC). Validation performed via cross-validation and real-time decision-making simulation in a web-based system. | Not specified |
| Chen (2025) | Treatment | Single-cell RNA sequencing (scRNA-seq), bulk transcriptomics, and genome-wide association studies (GWAS) from multiple LUAD patient cohorts. | China | Lung cancer | Lung adenocarcinoma (LUAD). Study used scRNA-seq, GWAS, and bulk transcriptomics to classify LUAD into two subtypes and assess immune infiltration. | - | Lung adenocarcinoma (LUAD). Validation performed using independent LUAD datasets. | Not specified |
| Pan & Wang (2025) | Treatment | Clinical data from 274 patients at Henan Cancer Hospital, China. | China | Liver cancer | 274 patients. 69.7% male, 30.3% female. 56.6% under 60 years, 43.4% aged 60+. Hepatocellular carcinoma (HCC). Patients received PD-1 or CTLA4 inhibitors; immune cell data and HBV DNA levels were analyzed. | - | Hepatocellular carcinoma (HCC). Validation performed via ten-fold cross-validation and Decision Curve Analysis (DCA). | Not specified |
| Chufal (2025) | Treatment | Clinical and DIBH assessment data from Rajiv Gandhi Cancer Institute & Research Centre, India. | India | Breast cancer | 202 patients. Breast cancer (left-sided). Included breath-hold duration, amplitude levels, and consistency parameters from DIBH assessment. | - | 47 patients. Breast cancer (left-sided). External validation performed with a prospective dataset. | Not specified |
| Miyamoto (2025) | Treatment | CT imaging data from Kumamoto University Hospital, Japan. | Japan | Colorectal liver metastases (CRLM) | 112 patients. 59% male, 41% female. Median: 64 years (range: 33–86). Colorectal liver metastases (CRLM). Patients treated with oxaliplatin- or irinotecan-based doublet chemotherapy; response classified using RECIST 1.1 criteria. | - | 38 patients. Colorectal liver metastases (CRLM). External validation using independent dataset. | JSPS KAKENHI (Grant No. 20K07702); Japanese Foundation for Multidisciplinary Treatment of Cancer; Tateishi Science and Technology Foundation. |
| Grosu (2025) | Treatment | CT colonography images from The Cancer Imaging Archive (TCIA). | Germany | Colorectal cancer | 59 patients, 77 polyps, 118 polyp image series. Colorectal polyps (adenomatous, non-adenomatous). CT colonography images segmented for AI-based classification. | - | Colorectal polyps. Model validated using histopathological reports as ground truth. | FöFoLe, Faculty of Medicine, Ludwig-Maximilians-University Munich, Germany. |
| Huang (2025) | Treatment | Electronic health records (EHR) and paper medical records from Hualien Tzu Chi General Hospital, Taiwan. | Taiwan | Oral cavity cancer | 168 patients. 90% male, 10% female. Oral cavity cancer. Patients underwent surgery followed by adjuvant radiotherapy with or without chemotherapy. | 19 patients. Oral cavity cancer. Not explicitly stated | Oral cavity cancer. Model validation performed via cross-validation. | Hualien Tzu Chi Hospital, Buddhist Tzu Chi Medical Foundation (TCRD-110-15, IMAR-110-01-08, TCRD112-032, TCRD112-047, TCMF-IMC 112-02, TCMJ-MP 113-01-01). |
| Zhang (2025) | Treatment | Surveillance, Epidemiology, and End Results (SEER) 18 database. | China | Head and neck squamous cell carcinoma (LA-HNSCC) | 7,376 patients. 82.8% male, 17.2% female. Median: 60 years (IQR: 53–67). Head and neck squamous cell carcinoma (LA-HNSCC). Patients received either concurrent CRT (5,326 patients) or surgery with postoperative RT/CRT (2,050 patients). | - | Head and neck squamous cell carcinoma (LA-HNSCC). Validation performed via inverse probability treatment weighting (IPTW). | National Natural Science Foundation of China (Grant No. 81873715). |
| Ramasamy (2024) | Treatment | Clinical and radiomics data from patients treated at Jewish General Hospital, Montreal, Canada. | Canada | Lung cancer | 98 patients, 116 lesions. 46% male, 52% female. Median: 76 years (Range: 35–94). Non-small cell lung cancer (NSCLC) (early-stage and oligometastatic). Patients received SBRT; radiomics extracted from CT scans; response classified using RECIST 1.1 criteria. | - | Non-small cell lung cancer (NSCLC). Cross-validation approach used to validate models. | Medteq Innovation [10-30 AI Multicentrique] |
