# Supplementary material for: Methodological and reporting quality of machine learning studies on cancer diagnosis, treatment, and prognosis
Source: Front Oncol. 2025 Apr 14;15:1555247. doi: 10.3389/fonc.2025.1555247 (PMC12034563; doi:10.3389/fonc.2025.1555247)
Supplement: Supplementary Table 2 — List of records included. [file Table2.docx]

**Supplementary Table 2.** List of records included.

| **TITLE** | **First author (year)** | **DOI** | **Type** |
| --- | --- | --- | --- |
| Development, validation, and clinical application of a machine learning model for risk stratification and management of cervical cancer screening based on full-genotyping hrHPV test (SMART-HPV): a modelling study | Dong (2025) | 10.1016/j.lanwpc.2025.101480 | Prognosis |
| Machine learning insights into early mortality risks for small cell lung cancer patients post-chemotherapy | Liang & Luo (2025) | 10.3389/fmed.2025.1483097 | Prognosis |
| Postoperative fever following surgery for oral cancer: Incidence, risk factors, and machine learning model | Zhang et al. (2025) | 10.1186/s12903-025-05555-9 | Prognosis |
| Leveraging survival analysis and machine learning for accurate prediction of breast cancer recurrence and metastasis | Noman (2025) | 10.1038/s41598-025-87622-3 | Prognosis |
| Machine learning based models for predicting presentation delay risk among gastric cancer patients | Zhou (2025) | 10.3389/fonc.2024.1503047 | Prognosis |
| Development of metastasis and survival prediction model of luminal and non-luminal breast cancer with weakly supervised learning based on pathomics | Liu (2025) | 10.7717/peerj.18780 | Prognosis |
| Development of a Predictive Model of Occult Cancer After a Venous Thromboembolism Event Using Machine Learning: The CLOVER Study | Franco-Moreno (2025) | 10.3390/medicina61010018 | Prognosis |
| Harnessing Machine Learning and Nomogram Models to Aid in Predicting Progression-Free Survival for Gastric Cancer Patients Post-Gastrectomy with Deficient Mismatch Repair (dMMR) | Li (2025) | 10.1186/s12885-025-13542-0 | Prognosis |
| Optimal Machine Learning Models for Developing Prognostic Predictions in Patients With Advanced Cancer | Hamano (2025) | 10.7759/cureus.76227 | Prognosis |
| Deep learning-based CT radiomics predicts prognosis of unresectable hepatocellular carcinoma treated with TACE-HAIC combined with PD-1 inhibitors and tyrosine kinase inhibitors | Yin (2025) | 10.1186/s12876-024-03555-7 | Prognosis |
| Construction of a prognostic prediction model for colorectal cancer based on 5-year clinical follow-up data | Xiao (2025) | 10.1038/s41598-025-86872-5 | Prognosis |
| Predicting the risk of gastroparesis in critically ill patients after CME using an interpretable machine learning algorithm – a 10-year multicenter retrospective study | Liu (2025) | 10.3389/fmed.2024.1467565 | Prognosis |
| CT-based Machine Learning Radiomics Modeling: Survival Prediction and Mechanism Exploration in Ovarian Cancer Patients | Su (2025) | 10.1016/j.acra.2024.12.047 | Prognosis |
| Personalized treatment strategies for breast adenoid cystic carcinoma: A machine learning approach | Alshwayyat (2025) | 10.1016/j.breast.2025.103878 | Prognosis |
| Development of a machine learning-based predictive model for transitional cell carcinoma of the renal pelvis in White Americans: A SEER-based study | Liu (2025) | 10.21037/tau-24-385 | Prognosis |
| Machine learning-assisted cancer diagnosis in patients with paraneoplastic autoantibodies | Maleki (2025) | 10.1007/s12672-025-01836-5 | Diagnosis |
| Machine-Learning Parsimonious Prediction Model for Diagnostic Screening of Severe Hematological Adverse Events in Cancer Patients Treated with PD-1/PD-L1 Inhibitors: Retrospective Observational Study by Using the Common Data Model | Park (2025) | 10.3390/diagnostics15020226 | Diagnosis |
| Enhancing breast cancer diagnosis using deep learning and gradient multi-verse optimizer: a robust biomedical data analysis approach | EL kati (2024) | 10.7717/peerj-cs.2578 | Diagnosis |
| MRI-based deep learning radiomics to differentiate dual-phenotype hepatocellular carcinoma from HCC and intrahepatic cholangiocarcinoma: a multicenter study | Wu (2025) | 10.1186/s13244-025-01904-y | Diagnosis |
| Artificial intelligence-assisted platform performs high detection ability of hepatocellular carcinoma in CT images: an external clinical validation study | Shan (2025) | 10.1186/s12885-025-13529-x | Diagnosis |
| An early lung cancer diagnosis model for non-smokers incorporating CT imaging analysis and circulating genetically abnormal cells (CACs) | Ni (2025) | 10.1186/s12885-024-13268-5 | Diagnosis |
| Diagnostic Power of MicroRNAs in Melanoma: Integrating Machine Learning for Enhanced Accuracy and Pathway Analysis | Rafiepoor (2025) | 10.1111/jcmm.70367 | Diagnosis |
| Blood Cancer Prediction Model Based on Deep Learning Technique | Shehta (2025) | 10.1038/s41598-024-84475-0 | Diagnosis |
| AI-Driven Enhancement of Skin Cancer Diagnosis: A Two-Stage Voting Ensemble Approach Using Dermoscopic Data | Chiu (2025) | 10.3390/cancers17010137 | Diagnosis |
| Construction of Prediction Model of Early Glottic Cancer Based on Machine Learning | Wang (2025) | 10.1080/00016489.2024.2430613 | Diagnosis |
| Boosting Skin Cancer Diagnosis Accuracy with Ensemble Approach | Natha (2025) | 10.1038/s41598-024-84864-5 | Diagnosis |
| Breast Tumor Detection and Diagnosis Using an Improved Faster R-CNN in DCE-MRI | Gui (2024) | 10.3390/bioengineering11121217 | Diagnosis |
| Diagnostic Performance of Deep Learning Applications in Hepatocellular Carcinoma Detection Using Computed Tomography Imaging | Şahin (2025) | 10.5152/tjg.2024.24538 | Diagnosis |
| Development of Machine Learning Models for Diagnostic Biomarker Identification and Immune Cell Infiltration Analysis in PCOS | Chen (2025) | 10.1186/s13048-024-01583-1 | Diagnosis |
| Quantitative Histopathology Analysis Based on Label-free Multiphoton Imaging for Breast Cancer Diagnosis and Neoadjuvant Immunotherapy Response Assessment | Zhong (2025) | 10.7150/ijbs.102744 | Diagnosis |
| Integration of MRI radiomics and clinical data for preoperative prediction of vascular invasion in breast cancer: A deep learning approach | Pan (2025) | 10.1016/j.mri.2025.110339 | Treatment |
| Establishing a preoperative predictive model for gallbladder adenoma and cholesterol polyps based on machine learning: a multicentre retrospective study | Wang (2025) | 10.1186/s12957-025-03671-y | Treatment |
| Integrating traditional machine learning with qPCR validation to identify solid drug targets in pancreatic cancer: a 5-gene signature study | Wang (2025) | 10.3389/fphar.2024.1539120 | Treatment |
| Artificial intelligence can help individualize Wilms tumor treatment by predicting tumor response to preoperative chemotherapy | Nashat (2025) | 10.4111/icu.20240135 | Treatment |
| Machine Learning Based on Blood Test Biomarkers Predicts Fast Progression in Advanced NSCLC Patients Treated with Immunotherapy | Zhou (2024) | 10.1136/bmjonc-2023-000128 | Treatment |
| Predicting the Effectiveness of Chemotherapy Treatment in Lung Cancer Utilizing Artificial Intelligence-Supported Serum N-Glycome Analysis | Torok (2025) | 10.1016/j.compbiomed.2025.109681 | Treatment |
| Enhancing Treatment Decisions for Advanced Non-Small Cell Lung Cancer with Epidermal Growth Factor Receptor Mutations: A Reinforcement Learning Approach | Bozcuk (2025) | 10.3390/cancers17020233 | Treatment |
| Integrative Multi-Omics Analysis for Identifying Novel Therapeutic Targets and Predicting Immunotherapy Efficacy in Lung Adenocarcinoma | Chen (2025) | 10.20517/cdr.2024.91 | Treatment |
| Antiviral therapy can effectively suppress irAEs in HBV positive hepatocellular carcinoma treated with ICIs: validation based on multi machine learning | Pan & Wang (2025) | 10.3389/fimmu.2024.1516524 | Treatment |
| Machine learning model for predicting DIBH non-eligibility in left-sided breast cancer radiotherapy: Development, validation, and clinical impact analysis | Chufal (2025) | 10.1016/j.radonc.2025.110764 | Treatment |
| Radiomics-based Machine Learning Approach to Predict Chemotherapy Responses in Colorectal Liver Metastases | Miyamoto (2025) | 10.23922/jarc.2024-077 | Treatment |
| Effect of Artificial Intelligence-Aided Differentiation of Adenomatous and Non-Adenomatous Colorectal Polyps at CT Colonography on Radiologists’ Therapy Management | Grosu (2025) | 10.1007/s00330-025-11371-0 | Treatment |
| Using a Machine Learning Algorithm and Clinical Data to Predict the Risk Factors of Disease Recurrence After Adjuvant Treatment of Advanced-Stage Oral Cavity Cancer | Huang (2025) | 10.4103/tcmj.tcmj_56_24 | Treatment |
| Individualized Treatment Recommendations for Patients with Locally Advanced Head and Neck Squamous Cell Carcinoma Utilizing Deep Learning | Zhang (2025) | 10.3389/fmed.2024.1478842 | Treatment |
| Models and Biomarkers for Local Response Prediction in Early-Stage and Oligometastatic Non-small Cell Lung Cancer Patients Treated With Stereotactic Body Radiation Therapy Using Machine Learning | Ramasamy (2024) | 10.7759/cureus.75819 | Treatment |
